# Aortic aneurysm management results through one year with a conformable neck sealing endograft and preemptive sac embolization with shape memory polymer devices

**DOI:** 10.1016/j.jvscit.2024.101656

**Published:** 2024-10-22

**Authors:** Dipankar Mukherjee, Jihui Li, David Spinosa

**Affiliations:** a Department of Surgery, Vascular Surgery, Inova Fairfax Medical Campus, Fairfax; b Advanced 3D Technology Lab, Inova Fairfax Hospital, Fairfax; c Department of Interventional and Diagnostic Radiology, Inova Fairfax Medical Campus, Fairfax

**Keywords:** Endovascular aneurysm repair, Endoleak prevention, Sac shrinkage

## Abstract

Management of abdominal aortic aneurysm includes reducing the incidence of endoleaks and promoting sac regression. Sac embolization has been shown to promote regression but alone may not adequately address type II endoleak risk. We present three cases with challenging anatomy and follow-up data through 12 months after treatment. Patients were treated with endografts, and shape memory polymer embolization plugs were used to embolize the residual lumen volume outside of the endograft during the procedure. Follow-up imaging indicates that this procedure was used successfully in these patients. None of the patients developed sac expansion or developed type II endoleaks.

Type II endoleaks are a consideration when managing abdominal aortic aneurysms (AAAs) via endovascular aneurysm repair (EVAR). These complications contribute to aneurysm failure to regress, which is linked to lower long-term survival.^1^ Risk factors include presence of large, patent sac feeding vessels, including inferior mesenteric artery (IMA), lumbar arteries (LAs), and accessory renal or sacral arteries.^2^^,^^3^ Outcomes vary in reports of IMA and sac embolization with coils and fibrin glue.^4^, ^5^, ^6^

Anatomical challenges like angulated and/or short necks and narrow common iliac arteries (CIAs) preclude many patients, especially women, from EVAR eligibility.^7^^,^^8^ Lower-profile devices expand treatment options but are associated with higher type II endoleak rates than standard stent grafts.^9^, ^10^, ^11^

Shape memory polymer (SMP) is a novel vessel embolization technology^12^, ^13^, ^14^, ^15^, ^16^ with several properties amenable to AAA sac management. IMPEDE-FX Embolization Plugs (Shape Memory Medical) incorporate SMP, a porous, thrombotic structure when expanded in a vessel. These devices have been shown in preclinical studies to promote cellular growth as the polymer gradually degrades with low incidence of recanalization (Fig 1).^14^^,^^17^

**Fig 1:**
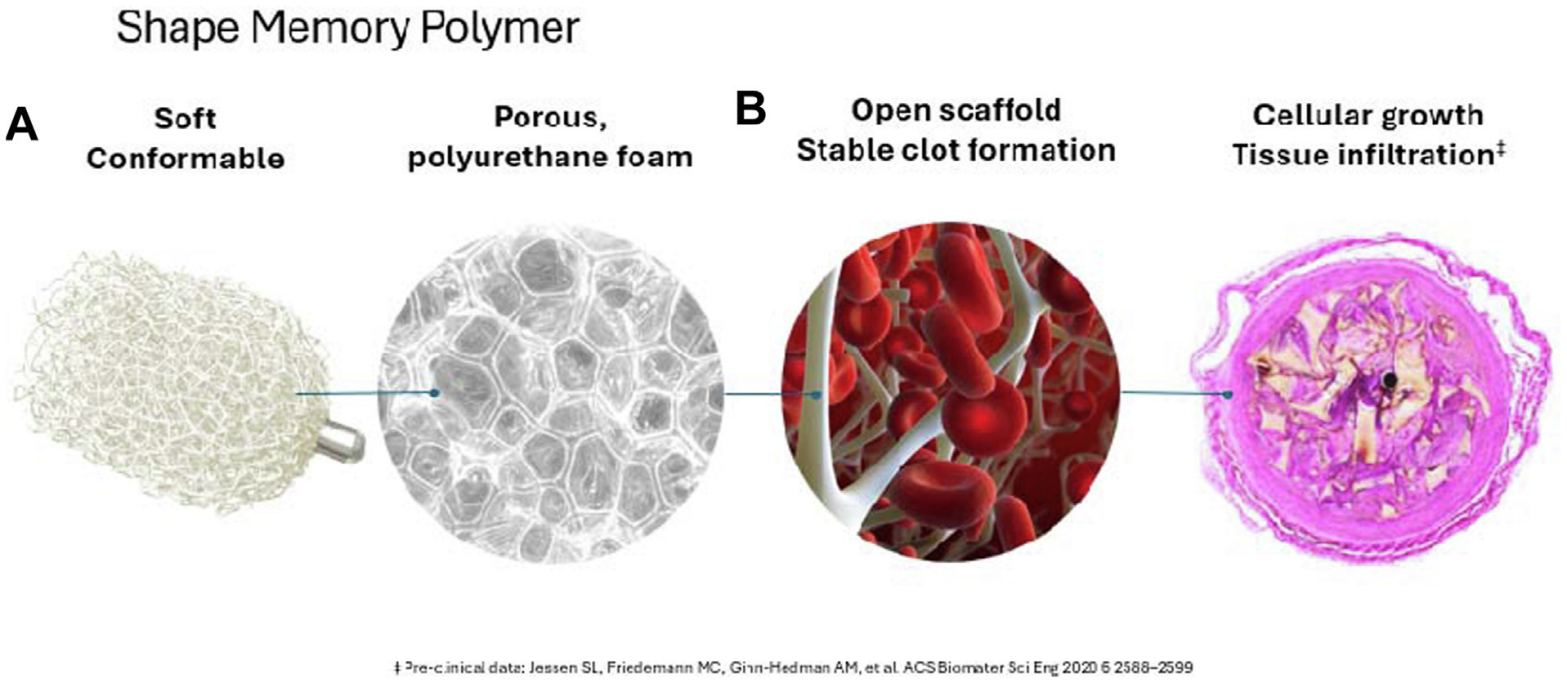
Shape memory polymer (SMP) embolization device used in all cases. **(A)** The device is crimped for catheter delivery; **(B)** The device self-expands on contact with blood to form a porous scaffold. Each device occupies approximately 1.25 mL when fully expanded. The radiopaque marker facilitates delivery and tracking in the sac and on follow-up imaging. The SMP itself is radiolucent. Image provided courtesy of Shape Memory Medical, Inc.

We present three AAA sac management cases with SMP and the low-profile ALTO abdominal stent graft system (Endologix) without complication in patients with challenging anatomy, who provided consent for publication. Featuring an aortic body with a network of polymer-filled sealing rings for aneurysm exclusion and flexible limbs, the ALTO system is suited for challenging femoral artery access and iliac artery tortuosity^10^ (Fig 2). These patients were also at increased risk of type II endoleak due to presence of large, patent LAs and IMAs. We embolized the AAA sac with SMP devices immediately following ALTO placement. The SMP devices were deployed within the aneurysm lumen, around the endograft, to promote sac thrombosis and feeding vessel occlusion using previously described methods.^13^ Fill volume were estimated using preoperative computed tomography angiogram (CTA) when available, or intraoperative sacograms, with each SMP device filling 1.25 ml. Following sac embolization, no residual lumbar artery filling was observed upon completion angiography. We evaluated sac regression and endoleak metrics through 12 months.

**Fig 2:**
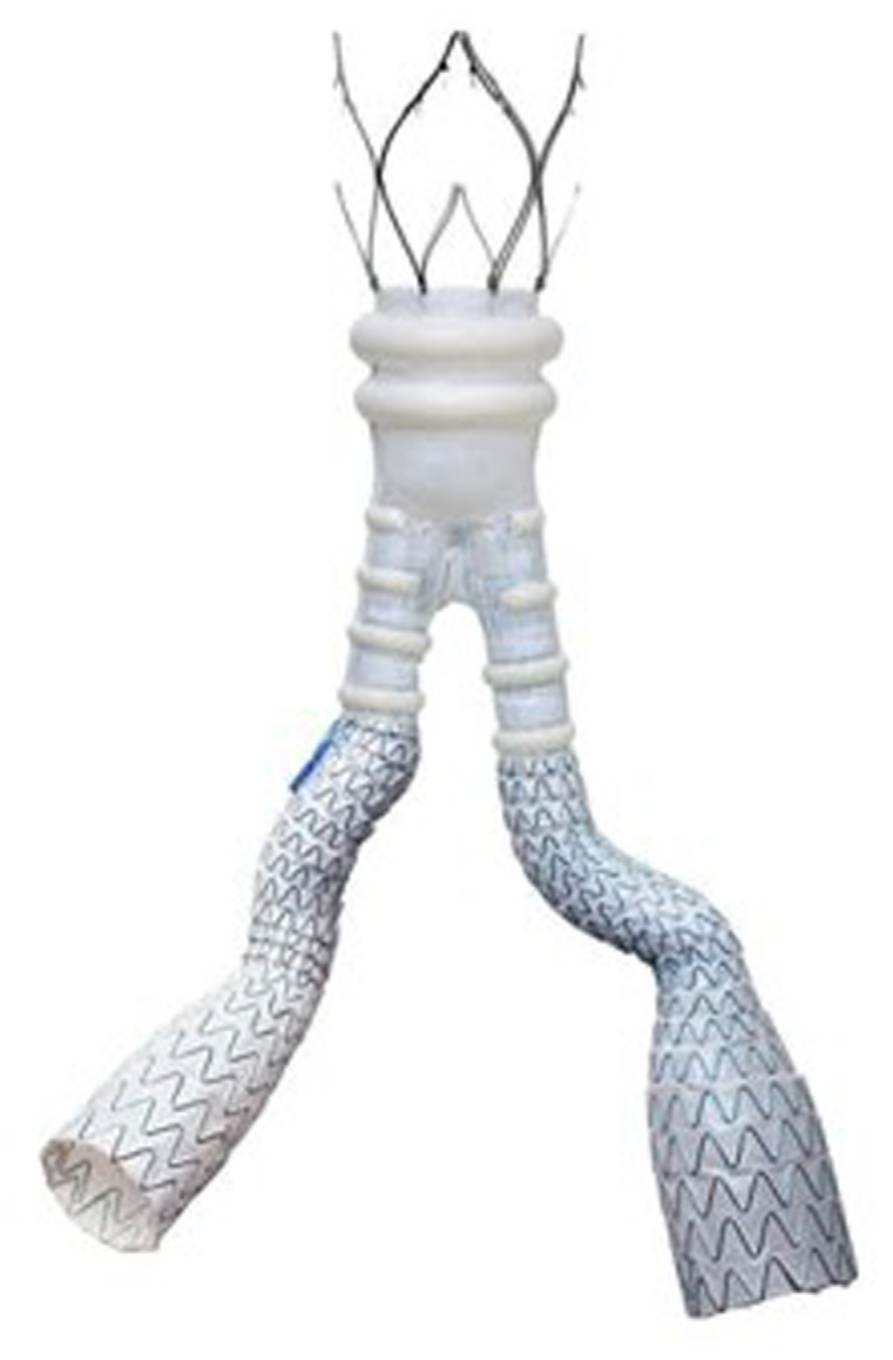
Endologix Alto abdominal stent graft.

Patient and preprocedural aneurysm characteristics are listed in Table. Standard procedures are described in the Supplementary Material (online only).

**Table:** Case characteristics

Characteristic	Case 1	Case 2	Case 3
Sex	Female	Female	Male
Age, years	73	62	82
AAA diameter	55	51	70
IMA diameter, mm	3	Occluded	Occluded
Patent lumbar arteries	Yes	Yes	Yes
Aberrant right renal artery	No	No	No
Iliac artery occlusion	Yes	No	Yes, right
Infrarenal neck length, mm	46.5	22	47.3
Neck angle, °	48.2	17	60
Estimated aneurysm volume, mL^a^	86.8	88.9	196.6
Endograft dimensions			
Main body, mm^b^	26	20	29
Left limb, mm^c^	14 × 120	10 × 100	14 × 160
Right limb^c^	14 × 120	12 × 120	14 × 160
SMP devices^d^			
Number of devices, No.	30	50	36
Fully expanded volume, mL^e^	37.5	62.5	45

*AAA,* Abdominal aortic aneurysm; *IMA,* inferior mesenteric artery; *SMP,* shape memory polymer.

a Based on preprocedural computed tomography angiography.

b Diameter. ALTO abdominal stent graft system (Endologix).

c Diameter x length. Ovation IX limb (Endologix).

d 12-mm diameter IMPEDE-FX Embolization Plug (Shape Memory Medical).

e Each SMP device self-expands to occupy a volume of ∼1.25 mL.^14^

## Case reports

### Case 1 procedure

Due to small iliac access vessels, the ALTO endograft was chosen. It was positioned at the right renal artery lower border and anchored by top crown deployment. The endograft crossover technique for wire and sheath placement was employed in preparation for contralateral limb deployment, as described previously.^13^

**Fig 3:**
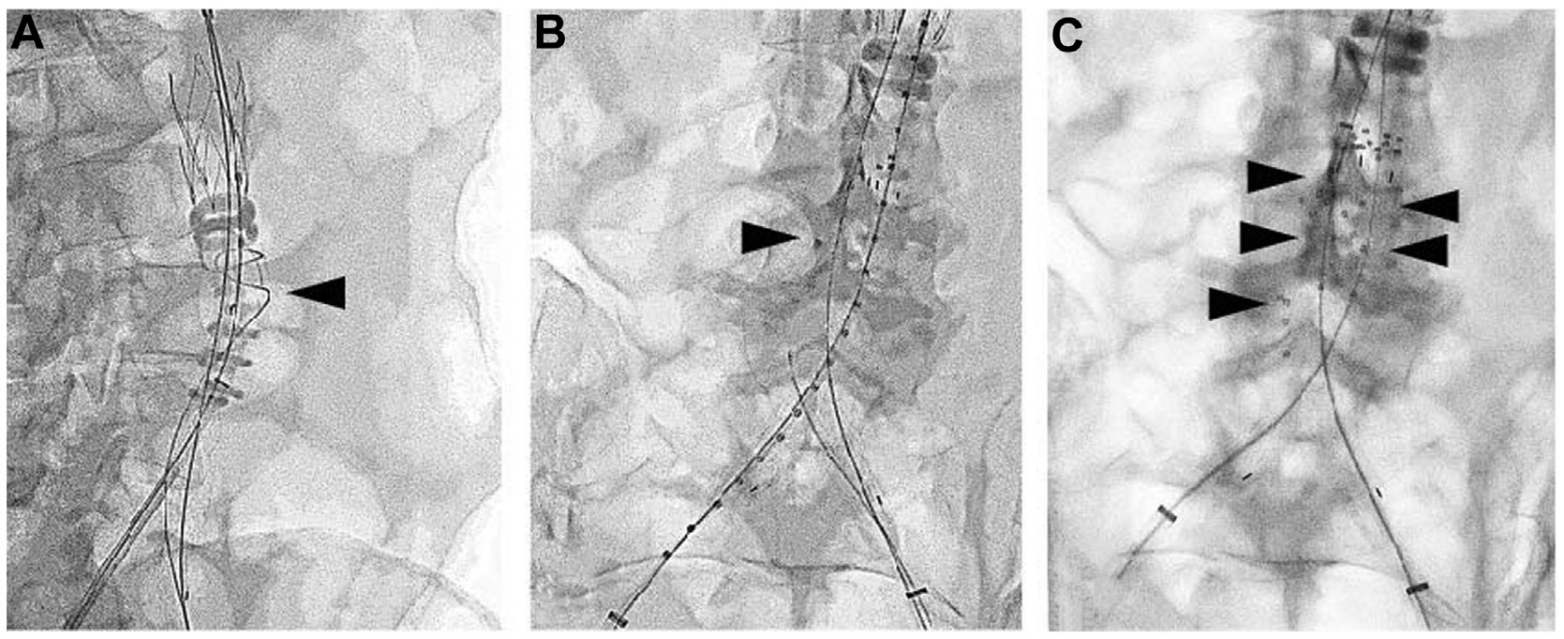
Delivery of shape memory polymer (SMP) devices. **(A)** Intraoperative radiograph showing endograft main body placement with guidewire access (*arrow*) into the aneurysm sac for Impede-FX plug placement; **(B)** Delivery of Impede-FX plug into aneurysm sac via vascular sheath and pushing guidewire; **(C)** Proximal markers of Impede-FX plugs in aneurysm sac.

With the sac isolated, 15 SMP devices were delivered into the residual flow lumen, concentrated near the large LA ostia in the upper sac region (Fig 3, *A-C*). An additional 15 SMP devices were placed in the capacious lower sac region for a total filling volume of 37.5 ml. Angiography after SMP delivery demonstrated thrombosis with minimal, non-pulsatile residual flow from the delivery sheath. The guiding sheath was removed and both limbs were balloon-sealed using a kissing technique, particularly at the flow divider and distal seal zones.

**Fig 4:**
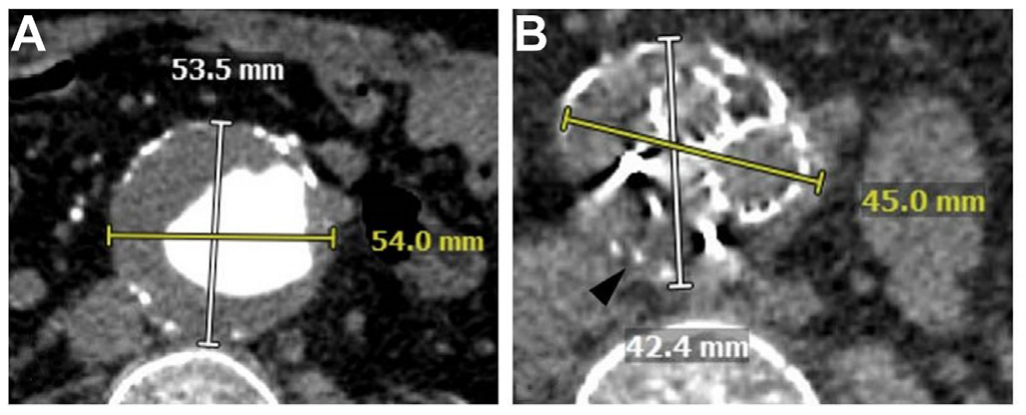
Pre-procedural and post-procedural imaging for Case 1. **(A)** Pre-procedural computed tomography angiography (CTA) showing 5.4 cm infrarenal abdominal aortic aneurysm; **(B)** One-year follow up CT shows continued sac diameter reduction. Metal artifacts from plug markers are seen.

Angiography showed total aneurysm exclusion with no endoleak. The patient was procedurally anti-coagulated, potentially contributing to observation of transgraft contrast blush. One-year follow-up CTA showed no endoleak evidence and continued sac diameter reduction (Fig 4, *A-B*).

### Case 2 procedure

The patient had an area of slight outpouching in the proximal neck below the left renal artery. Due to the type IA endoleak risk, the ALTO endograft was chosen. The endograft was deployed at the left renal artery lower border, with proximal seal confirmed via angiography. The endograft crossover wire technique snared the wire from left to right, and a limb was deployed extending proximally between the third and fourth ring of the aortic body and distally in the left CIA. A guidewire was placed in the sac, the endograft limb was deployed, and the SMP delivery sheath was advanced into the sac over the jailed wire to deliver the SMP plugs.^13^ Fifty devices, providing 62.5 ml of fill volume were delivered into the residual flow lumen within the lower region of the sac, close to the narrow aortic bifurcation. The delivery sheath was reoriented, and additional devices were advanced toward the lumbar arteries, eliminating outflow as shown via angiography. The guiding sheath was removed and the endograft limbs re-ballooned, confirming distal seal.

Angiography revealed good bilateral filling of external and internal iliac arteries and total aneurysm exclusion with no evidence of endoleak post-procedurally or at six-month CTA. At 1-year follow-up, color-enhanced DUS showed a 1.0 cm diameter regression to 4.1 cm from 5.1 cm and no endoleak.

### Case 3 procedure

This patient had a large infrarenal AAA and bilateral iliac artery aneurysms, complicated by extremely tortuous iliac arteries, challenging access, and chronic renal insufficiency limiting contrast use. Following standard access, the patient briefly experienced hypotension as the 11 French sheath was placed on the right side. Angiogram through the sheath side arm revealed extravasation from the distal external iliac artery. The common and external iliac areas were lined with Gore Viabahn and VBX stents (Gore Medical, Inc), extravasation absence was confirmed by angiography, and the patient remained hemodynamically stable. The main body was placed at the right renal artery lower border, with 035 Bentson wire placed in the sac followed by the Iliac limb, placed prior to SMP delivery sheath insertion.^13^

Thirty-six SMP devices (45 ml fill volume) were delivered using bilateral access^13^ for complete sac thrombosis. The iliac limbs were re-ballooned in a kissing fashion from the flow divider through both limbs with particular attention to the distal landing zones. Completion angiography showed aneurysm exclusion with no evidence of endoleak. Twelve-month CEUS showed no endoleak and sac regression.

## Discussion

These cases highlight the use of a low-profile endograft in patients with challenging AAA neck and iliac anatomy and type II endoleak risk. They also demonstrate the introduction of a novel sac management technology, delivered via vascular sheath in the sac contralaterally (Cases 1-2) and bilaterally (Case 3) without the addition of clinically significant time or substantial cost.^18^^,^^19^ Early in our experience, we used contralateral access, but we have evolved to using a bilateral approach, and think it is an improvement. Currently, we place two 6 French destination sheaths deep into the aneurysm sac over Benston wires and deploy embolization plugs via both sheaths as the sheaths are withdrawn from caudal to cranial orientation within the aneurysm sac.

Coil embolization for both surgical and endovascular AAA repair has been described with some success in preventing type II endoleaks.^5^^,^^6^^,^^20^^,^^21^ The volume-filling properties of SMP in sac exclusion are intriguing, particularly the material’s radiolucency with minimal imaging artifact when monitoring for endoleaks. The feasibility of sac management using this novel technology has been reported with clear sac regression up to 1-year post-procedure.^12^^,^^15^ Our experience complements these findings and emphasizes the need for adequately filling the residual flow lumen volume, particularly in large aneurysms. EVAR with SMP sac management, rather than EVAR alone, may yield superior results going forward.

## Conclusion

In these three cases, the use of an ultra-low profile endograft in combination with SMP devices enabled treatment of patients with complex anatomy, and preemptively addressed type II endoleak risk. Larger studies with longer-term follow-up will establish the efficacy of this approach for sac regression and endoleak prevention.
