# Supplementary material for: Aortic aneurysm management results through one year with a conformable neck sealing endograft and preemptive sac embolization with shape memory polymer devices
Source: J Vasc Surg Cases Innov Tech. 2024 Oct 22;11(1):101656. doi: 10.1016/j.jvscit.2024.101656 (PMC11626527; doi:10.1016/j.jvscit.2024.101656)
Supplement: Supplementary Material (online only) [file mmc1.docx]

**Supplemental Materials**

**Standard Procedures**

***Access***

Bilateral common femoral artery access was achieved by ultrasound-guided percutaneous puncture (Merit Medical, Inc., South Jordan, Utah, USA). A dilator and sheath were placed over 0.018” wires (Cook Incorporated, Bloomington, Indiana, USA), which were replaced by a 0.035” Bentson wire (Cook Incorporated, Bloomington, Indiana, USA), and vessel closure sutures were put in place, as applicable. Bilateral endograft access sheaths were placed over the Bentson wires; on the main body access side (ipsilateral), the Bentson wire was exchanged via an exchange catheter for a 0.035” stiff wire (Lunderquist^®^ Wire Guide, Cook Incorporated., Bloomington, Indiana, USA) and a marker pigtail catheter (Accu-Vu Pigtail, Angiodynamics, Inc., Latham, New York, USA) was introduced on the contralateral side. Note that there is need to upsize the sheath by two French sizes on the side contralateral to the main body to accommodate the sheath used for sac embolization. Similarly, if two sheaths are placed in the aneurysm sack, the ipsilateral sheath would have to be upsized by two French sizes as well.

***Endograft crossover wire technique***

A 0.014” wire was introduced through the crossover channel built into the endograft, snared from the contralateral side, and externalized through the contralateral femoral artery access point. A flush catheter was introduced over the externalized wire into the endograft limb and a 0.014” buddy wire (Hi-Torque Spartacore^™^ Abbott Vascular, Santa Clara, California, USA) was advanced into the suprarenal aorta. The crossover wire was then withdrawn from the original side, the flush catheter was advanced into the suprarenal aorta, and the buddy wire was exchanged for a standard sized Amplatz wire (Boston Scientific, Inc., Marlborough, Massachusetts, USA).

***Endograft limb sizing***

The endograft limb lengths were determined based on the distance from the flow divider to the distal common iliac artery.

***Estimation of Aneurysm Volume and Number of SMP Plugs***

For these patients, sac volume size was estimated using standard intra-procedural imaging. Early in our experience we did not do sac volume measurements with 3D imaging as we do now, however, we estimated sac volume using standard CTA and angiography. We then delivered the SMP plugs and observed how each delivery filled the sac before delivering additional plugs. Note that the inner diameter of the sheath must be between .070” - .090” to be compatible with SMP plug delivery. This translates to most 5F or 6F introducer sheaths.

Each device fills 1.25 ml. As our experience grew and we began using more advanced imaging, we developed a better sense of the number of plugs needed for a given case.

***Vessel closure***

Sheaths were removed and access points were closed by cinching up on the vessel closure device sutures (Abbott Perclose^™^ ProStyle^™^ Abbott Cardiovascular, Inc., Plymouth, Minnesota, USA); or via other standard vessel closure devices (Gore^®^ DrySeal, Gore Medical, Inc., Flagstaff, Arizona, USA and Manta^®^ Vascular Closure Device, Teleflex, Inc., Morrisville, North Carolina, USA).
